# Convolutional Neural Networks for the Real-Time Monitoring of Vital Signs Based on Impulse Radio Ultrawide-Band Radar during Sleep

**DOI:** 10.3390/s23063116

**Published:** 2023-03-14

**Authors:** Sang Ho Choi, Heenam Yoon

**Affiliations:** 1 School of Computer and Information Engineering, Kwangwoon University, Seoul 01897, Republic of Korea; 2 Department of Human-Centered Artificial Intelligence, Sangmyung University, Seoul 03016, Republic of Korea

**Keywords:** IR-UWB radar, noncontact, vital-sign monitoring, real time, deep learning

## Abstract

Vital signs provide important biometric information for managing health and disease, and it is important to monitor them for a long time in a daily home environment. To this end, we developed and evaluated a deep learning framework that estimates the respiration rate (RR) and heart rate (HR) in real time from long-term data measured during sleep using a contactless impulse radio ultrawide-band (IR-UWB) radar. The clutter is removed from the measured radar signal, and the position of the subject is detected using the standard deviation of each radar signal channel. The 1D signal of the selected UWB channel index and the 2D signal applied with the continuous wavelet transform are entered as inputs into the convolutional neural-network-based model that then estimates RR and HR. From 30 recordings measured during night-time sleep, 10 were used for training, 5 for validation, and 15 for testing. The average mean absolute errors for RR and HR were 2.67 and 4.78, respectively. The performance of the proposed model was confirmed for long-term data, including static and dynamic conditions, and it is expected to be used for health management through vital-sign monitoring in the home environment.

## 1. Introduction

Interest in healthcare in daily life is increasing as the average life expectancy increases. With the global pandemic of COVID-19, an aging population, and an increase in the number of chronically ill people, the paradigm of global healthcare is gradually shifting from diagnosis and treatment to prevention, prediction, and personalization. Accordingly, various Internet of Things (IoT) and wearable devices are released into the healthcare industry, and new healthcare services are provided.

Health monitoring in a home environment is necessary to prevent and predict diseases, and manage health. In particular, technology that monitors vital signs is essential for emergency responses and stable healthcare. Respiration is an important vital sign for examining health conditions. Changes in physiological signals are associated with physical and physiological stress responses related to critical conditions. According to the World Health Organization, millions of people suffer from chronic respiratory problems. In addition, apnea and hypopnea, which occur during sleep, can be fatal to the elderly, overweight, and chronically ill, and can increase the risk of asthenia, cancer, and chronic kidney disease under certain conditions. Therefore, monitoring respiration can predict and consequently enable the prevention and treatment of respiratory diseases at an appropriate time.

Several technologies have been proposed to monitor cardiopulmonary signals; systems with high accuracy are typically in the form of skin contacts or wearable devices. Although these systems can be utilized in hospital environments that require precise measurements, they are inconvenient for long-term use in everyday home environments.

A contactless radio-frequency-based monitoring solution capable of solving these problems was studied and proposed [1,2,3,4,5,6,7]. This method has the advantage of being able to monitor the health of the user for a long period of time in an unconstrained manner. A radio signal from the radar is transmitted to the patient with a transceiver, and various pieces of information can be obtained on the basis of the characteristics of the modulation caused by the displacement of the chest wall. For example, devices based on radar technology were used in user count detection [8,9,10], localization [11,12], and gesture recognition [13,14]. The major types of radar employed in various fields include continuous wave (CW) [15], frequency-modulated continuous wave (FMCW) [16], and impulse radio ultrawide-band (IR-UWB) [17] radar; research has been conducted using these to monitor vital signs.

Shyu et al. [1] proposed the first valley peak of the energy function of the intrinsic two-layer mode-function-based ensemble empirical mode decomposition method to simultaneously extract heartbeat and breath information. Zhang et al. [2] introduced a harmonic multiple-loop detection algorithm for the noncontact detection of vital signs using IR-UWB radar. Their algorithm only needed to extract fundamental and second-harmonic components to estimate vital signs. The authors in [3] investigated the use of millimeter-wave FMCW radar to remotely monitor vital signs by employing advanced phase unwrapping manipulation, which is a unique method for extracting the vital signs of a subject lying down on a bed. Ren et al. [4] evaluated the performance of stepped-frequency CW radar and imaging photoplethysmography systems for remote vital-sign detection. Their results showed that the two systems had similar performance when extracting vital signs under ambient light conditions. Algorithms for the contactless monitoring of vital signs by utilizing radar have been proposed in several studies. However, in most previous studies, the distance between the radar and subject was fixed in a static state, and verification was performed using data from only a short time of measurements. To improve the utilization of radar technology, systems must be verified by employing real environmental data, including movement, and data that have been measured over a long period of time.

Radar technology is used not only to monitor vital signs, but also to detect apnea–hypopnea (AH) events during sleep. Choi et al. [5] demonstrated the feasibility of utilizing deep learning and an FMCW radar to detect sleep apnea–hypopnea events. In [6], the authors developed and verified an algorithm to detect obstructive sleep apnea events using an IR-UWB radar. Kwon et al. [7] proposed a hybrid convolutional neural network (CNN)–long short-term memory (LSTM) framework that could detect AH events in real time using an IR-UWB radar. They achieved state-of-the-art performance in classifying sleep apnea and hypopnea syndrome severity without any hand-engineered features. The continuous monitoring of vital signs during daily life can prevent an increase in the risk of cardiovascular disease due to the occurrence and exacerbation of sleep apnea and hypopnea syndrome (SAHS), and is also important in managing patients with SAHS. In addition to detecting AH events using radar technology for the SAHS group, the applicability of vital-sign estimation technology to the SAHS group needs to be examined.

In this study, we evaluated the estimation performance of a multidimensional CNN-based model for the real-time vital-sign detection using an IR-UWB radar. The performance of the proposed model was verified using long-term data measured during the static and dynamic states of sleep. Furthermore, we evaluated the model by employing SAHS patient data and compared its performance with that achieved in previous works.

## 2. Materials and Methods

### 2.1. Experimental Setup and Data Acquisition

In this study, polysomnography (PSG), the gold-standard method for evaluating sleep, was utilized; data were simultaneously measured using an IR-UWB radar. PSG is a standard test method employed to evaluate sleep and diagnose sleep-related diseases by measuring various biosignals during sleep, such as electroencephalography, electrooculography, electrocardiography (ECG), electromyography, respiration, and SpO2. Sleep physicians and sleep technicians score sleep stages and sleep breathing-disorder events according to the American Academy of Sleep Medicine manual [18] using these measured biosignals. In this study, the pressure transducer airflow (PTAF) signal measured with PSG was utilized as a respiration reference signal, and the ECG signal was employed as a heart-rate (HR) reference signal. The PTAF sensor measures the pressure difference between inhalation and exhalation using a nasal cannula, which is one of the main biosignals used to identify apnea and hypopnea events, and it is more sensitive to hypopnea than airflow or respiratory induction plethysmography is.

Assuming a daily home environment, the IR-UWB radar (Novelda, Oslo, Norway) was placed at 0.5–2 m from the bed, and the data were recorded simultaneously with PSG. The central frequency of the UWB radar transmitter was set to 7.3 GHz, and the bandwidth was set to 1.5 GHz; the sampling rate of the receiver was 23.328 GS/s, and the radar signal was digitized at 20 fps. To synchronize the data from PSG and the IR-UWB radar, subjects were instructed to hold their breath for approximately 15–20 s immediately after recording had begun, and the data from both systems were synchronized on the basis of this calibration period.

Subjects with various intensities of sleep apnea and hypopnea syndrome (SAHS) and without SAHS participated in this study; their demographical information is summarized in Table 1. We tested 30 participants at the Sleep Medicine Center of Seoul National University Hospital (SNUH). This retrospective study used PSG and UWB radar data obtained from SNUH and was conducted after receiving approval from the Institutional Review Board of Kwangwoon University (IRB no. 7001546-202209030-HR(SB)-009-05).

### 2.2. Data Preprocessing

**Figure 1:**
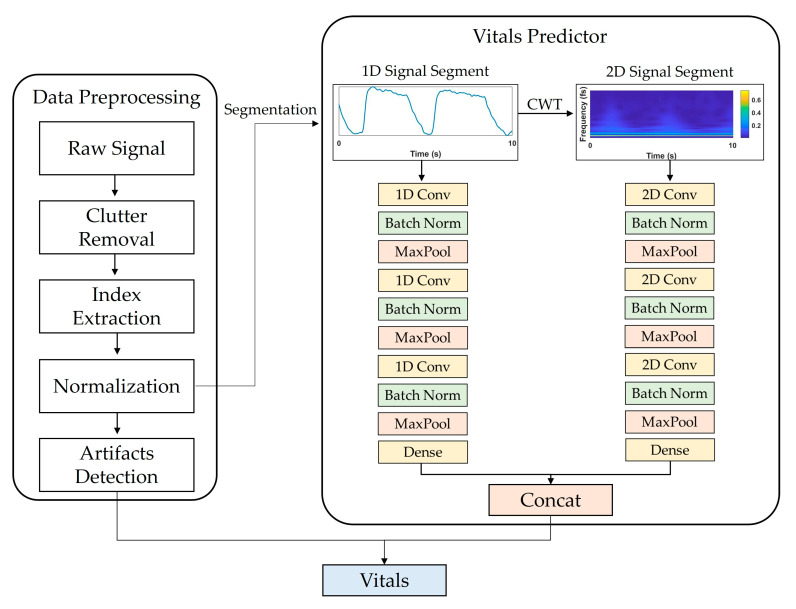
Overview of proposed DL model framework for estimating vital signs using IR-UWB radar signals. It consists of data preprocessing and vitals predictor parts. In data preprocessing, input data segments are extracted through clutter removal, index extraction, and normalization. In vitals prediction, the extracted segments are input into the CNN model that processes 1D and 2D signals, and RR and HR are extracted as final outputs.

Figure 1 shows a flowchart of the proposed system. Data preprocessing consisted of (1) IR-UWB radar signal processing, (2) target distance detection, and (3) artifact detection. The basic principle of extracting vital signs using IR-UWB is to measure the displacement caused by changes in the chest volume during breathing and for heartbeats. The activity of vital signs can be expressed as a mathematical model [19] of distance d over time t as follows:

(1) $$d\left(t\right)=d_{0}+m\left(t\right)=d_{0}+m_{r}\sin\left(2\pi f_{r}t\right)+m_{h}\sin\left(2\pi f_{h}t\right),$$

where $d_{0}$ represents the nominal distance between the subject and radar; $m_{r}$ and $f_{r}$ are the displacement amplitude and frequency of respiration, respectively; and $m_{h}$ and $f_{h}$ are the displacement amplitude and frequency of the heartbeat, respectively. The echo signals of a typical multichannel radar can be represented by the sum of the respiration, heartbeat, and noise components as $r\left(t,\;\tau \right),$ where t is a real-time factor (called the slow time), and τ is the observed range factor (called the fast time):

(2) $$r\left(t,\;\tau \right)=A_{d}p\left(\tau -\tau_{d}\left(t\right)\right)+\underset{i}{\sum }A_{i}p\left(\tau -\tau_{i}\right),$$

where $A_{d}$ and $\tau_{d}\left(t\right)$ represent the amplitude of the pulse reflected from the body and the corresponding time delay, respectively; $A_{i}$ is the amplitude of each multipath component; $\tau_{i}$ is the pulse delay; and $p\left(t\right)$ is the normalized received pulse. Echo signals $r\left(t,\;\tau \right)$ are then stored in Equation (3), where m and n represent discrete-time sequences and range frame size, respectively. $T_{s}$ is the slow time period, and $T_{f}$ is the sampling period.

(3) $$R\left[m,n\right]=r\left(t=mT_{s},\;\tau =nT_{f}\right)$$

**Figure 2:**
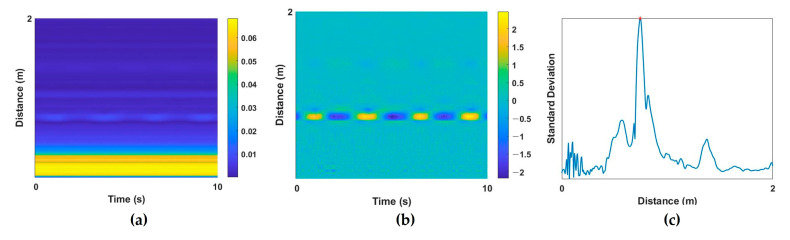
Example of preprocessing steps. UWB data (**a**) before and (**b**) after clutter removal; (**c**) standard deviation of the distance of each signal for target localization.

To extract vital signs from a raw radar signal, a multipath component called clutter should be removed. RF signals of IR-UWB radar are reflected not only by the desired target, but also by the objects within the radar range, and these unintended noise components are the clutter component. Background clutter components in a room include walls, furniture, floors, and objects. These elements produce static noise that appears as DC components. By removing these clutter components, human activity can be detected, and vital-sign information can be extracted. In this study, DC components were extracted from raw radar signals (Figure 2a) using the moving average method [20], and the corresponding clutter components were removed (Figure 2b). Then, the target index of the UWB signal was extracted for the next preprocessing step. When the subject is at a specific distance from the radar, the standard deviation (STD) of the received amplitude appears large at a specific slow time because of the displacements of periodic vital signs. On the basis of this characteristic, the index with the largest STD was set as the target index, and the radar signal at that index was extracted as a one-dimensional (1D) input signal. Next, z-score normalization was applied because the amplitude of the extracted 1D signal for each subject could differ. An example of the extracted target index based on the STD value is provided in Figure 2c.

Lastly, the motion and noise components of the extracted radar signals were detected. In the artifact section, the quality of the UWB signal is poor, and the vital signs may be buried in the noise; therefore, incorrect vital signs may be extracted. Thus, detecting these sections and maintaining the previous values of the vital signs or using interpolation can prevent abnormally estimated vital signs.

**Figure 3:**
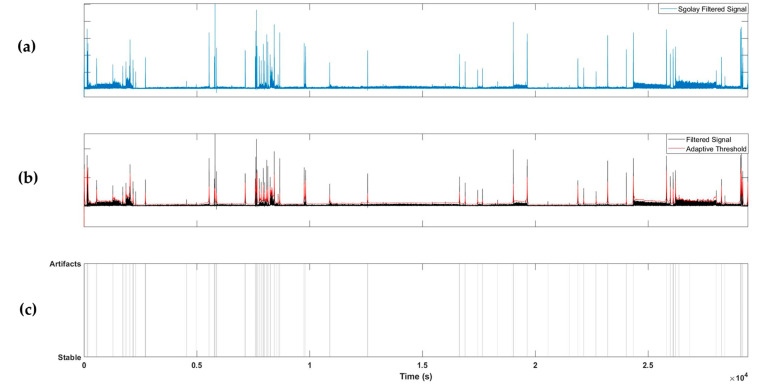
Example of artifact detection steps. (**a**) Applying Savitzky–Golay filter to the extracted radar signal; (**b**) applying an adaptive threshold; (**c**) labelling artifacts segments.

To detect artifacts, the absolute value of the 1D signal extracted from the radar was taken and filtered using a Savitzky–Golay FIR filter with a polynomial two-dimensional (2D) structure. The Savitzky–Golay filter [21] has a smoothing effect while protecting the peak shape characteristics of the signal and can be employed to detect transient artifacts. An adaptive threshold was determined using the filtered signal, and artifacts were detected if the value of the filtered signal exceeded the adaptive threshold. The adaptive threshold was calculated as the sum of the moving averages of the previously filtered signals with window sizes of 10, 30, and 100 s. Using this method, artifacts can be detected in both short and long periods of time; example results are shown in Figure 3.

### 2.3. Data Segmentation and Vitals Estimation

Data segmentation was performed for the deep learning model to learn the 1D radar signal extracted through preprocessing. First, filtering was applied to remove unnecessary artifact components other than vital signs. The extracted radar signals were high-pass-filtered with a 0.1 Hz cut-off frequency and then low-pass-filtered with a 5 Hz cut-off frequency (fifth infinite impulse response Butterworth filter).

The RR of a healthy person in a resting state is 12–20 respirations per minute (RPM) corresponding to a frequency range of 0.2–0.4 Hz. The resting HR is 60–100 beats per minute, corresponding to a frequency range of 1.0–1.7 Hz [22]. In biosignals, frequencies include various types of information; the fast Fourier transform (FFT) method can extract frequency information. In this study, a continuous wavelet transform (CWT) was applied to learn information from frequency changes over time in a deep learning model. The CWT [23] can extract the frequency components of time series signals, similar to FFT, and has the advantage of preserving time-domain information. A 2D signal in the time–frequency domain was extracted by applying CWT to the 1D time-series radar signal obtained during preprocessing. A Morse wavelet was utilized for CWT conversion, and the frequency limit was set to 0–5 Hz to focus on the frequency domain of the vital signs. A 2D signal was obtained by applying CWT to the 1D time-series radar signal obtained during preprocessing. A Morse wavelet was employed for CWT conversion, and the frequency limit was set to 0–5 Hz to focus on the frequency range of the vital signs.

After data segmentation, a dataset was formed, and the deep learning model was trained and tested. The window size was set to 15 s to include at least 2–3 breathing cycles, and segmentation was performed by moving the window by 1 s. When configuring the training dataset, segmentation was performed on the basis of a section without artifacts to prevent false learning due to artifacts, and a dataset for each 1D or 2D signal was configured. Considering real-time vital-sign estimation, the validation and test datasets were segmented, including artifact sections, and noise-containing sections were postprocessed. In this study, we trained and tested the model using the holdout method. From the 30 recordings, 10 recordings were used for training, 5 recordings were used for validation, and the remaining 15 recordings were used for the testing dataset; the employed recordings in each dataset were randomly selected. The total segments for the training, validation, and test datasets were 262,860, 135,945, and 599,595, respectively.

The goal of this study was to estimate the RR and HR using IR-UWB signals. The reference RR and HR values were extracted by detecting the peak and computing interpeak time using PTAF and ECG signals from PSG. Reference RR and HR values were utilized as labels for the dataset, and the model was trained by the supervised learning method.

### 2.4. Deep Learning Model Architecture

Figure 1 shows the vital-sign estimation network framework and the selected CNN architecture, which was configured to output RR and HR values by concatenating two independent CNN models that receive 1D and 2D signals as inputs and a fully connected layer.

To optimize the hyperparameters of the model, several CNN structures were trained and validated over a predetermined parameter range. After setting the candidate hyperparameters as follows, the optimal combination of parameters that produced the lowest mean absolute error (MAE) was taken to be the validation set. The hyperparameters of the searched model structure were the number of CNN layers {1,2,3}, number of convolutional filters {32,64,128}, and the CNN structure {1D, 2D, combined (1D + 2D)}. A total of 81 combinations of conv layer number, filter size, input dimensions, and window size existed, and the model with the lowest MAE was selected as the final model.

Batch normalization [24] was applied to each convolutional layer, the model was trained using the Adam optimizer [25], and the MAE was employed as the loss function. The learning rate was determined using the learning rate finder method [26], with a starting point of 1e–5. The total number of learning epochs was set to 50, and the early stopping method was applied to stop learning if the loss value did not decrease more than seven times in a row. The epoch model, which had the highest performance during learning, was saved. After learning was completed, the model weights were reverted to those of the best performance epoch, and this model was used for testing.

The Pytorch lightning (version 1.8.1) library [27] was used for model implementation, and model training was performed on a workstation equipped with a 3.4 GHz AMD Ryzen-5950X CPU, 64GB RAM, and two NVIDIA GeForce GTX3080ti 12GB GPUs.

## 3. Results

### 3.1. Proposed Model Performance

**Figure 4:**
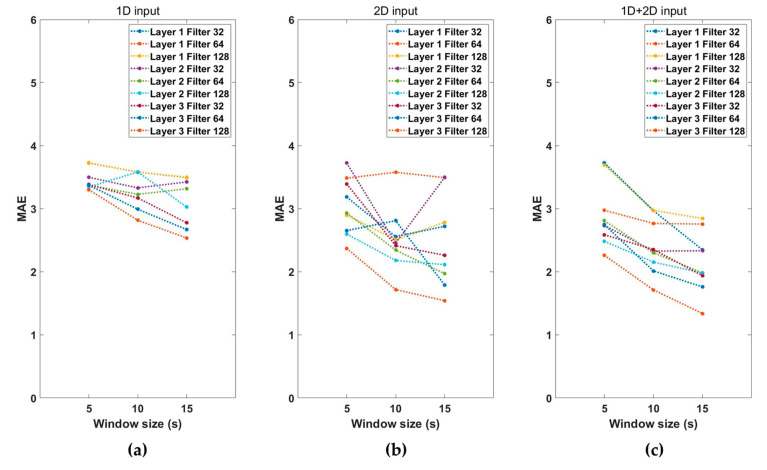
Performance of the (**a**) 1D input model, (**b**) 2D input model, and (**c**) 1D + 2D input model with the number of layers, filter size, and segment window size.

Performance according to the combination of hyperparameters for estimating the vital signs was examined. Figure 4 shows the MAE values according to the characteristics of the input signals, window sizes, number of convolution layers, and filter sizes. The MAE represents the sum of RR and HR estimation errors. As shown in Figure 4, the performance using both 1D and 2D input signals was better than that obtained using only 1D or 2D input data. The performance of the vital-sign estimation model increases as the window size increases, and the number of convolution layers and the number of filters increase. We then selected the hyperparameters combination model with the lowest MAE. The combination of hyperparameters selected as the final model used a 1D + 2D input signal, with a window size of 15 s three convolutional layers, and a filter size of 128. The MAE of the selected model was 1.34, and Table 2 shows the configuration of the final model.

### 3.2. Test Dataset Performance

**Figure 5:**
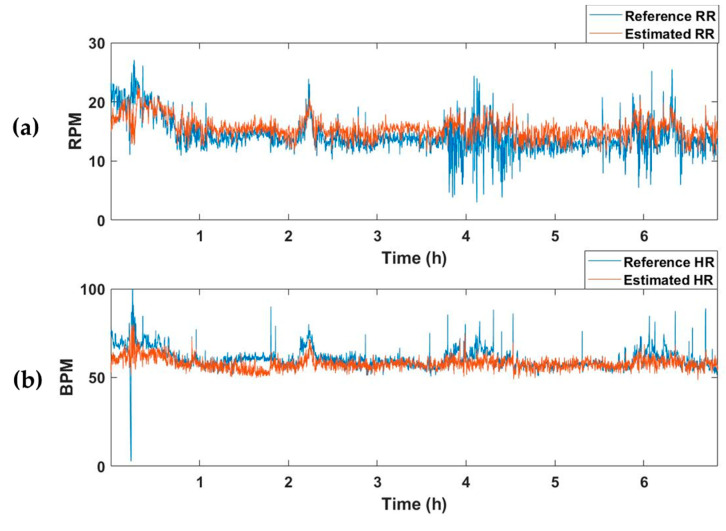
Example of the vital-sign detection results obtained using the vital-signs estimation model. (**a**) Reference and estimated RRs, and (**b**) reference and estimated HRs. RPM, respiration per minute; BPM, beats per minute.

The selected model with the best performance was applied to test dataset, which comprised 15 recordings. The test dataset was constructed by extracting segments while moving a 15 s window by 1 s. Table 3 summarizes the MAE values of RR and HR for the recordings of each subject. The average MAEs of RR and HR for all test datasets were 2.67 and 4.78, respectively. Figure 5 represents an example of the estimated vital-sign result for the entire sleep time of one subject. Figure 5a shows the reference RR and estimated RR, and Figure 5b depicts the reference and estimated HRs.

## 4. Discussion

Vital signs provide valuable information about the physical and mental health of a person. Therefore, monitoring vital signs in daily life provides a better understanding of health and alerts us to any changes that may require medical attention. To this end, this study proposed a real-time vital-sign monitoring deep learning framework using noncontact IR-UWB radar. The position of the subject was searched on the basis of STD values, and the selected 1D signal and a 2D signal applied with CWT were used as input signals. For each signal, CNN-based models were applied and concatenated to estimate vital-sign values in real time.

First, when comparing the performance according to the combination of hyperparameters of the model, the MAE of the combination of two dimensions was lower than that of sole 1D and 2D signals. This finding implies that extracting both the pattern characteristics of the 1D signal and the characteristics of the frequency components of the 2D signal extracts more information than considering only one type of signal does. The training results indicate that the data on 2D signals containing frequency components have more information in estimating vital signs than those of 1D signals with only time series information. Additionally, more information is extracted when time series signal pattern characteristic information is added to frequency component information.

Most previous studies on detecting vital signs using radar mainly utilized the frequency component information of radar signals [1,2,28,29,30,31]. Shyu et al. [1] proposed the ensemble empirical mode decomposition method to separate the heartbeat from respiration signal and employed FFT to extract RR and HR. The authors of [2] extracted RR and HR frequency bands using FFT and then input to the harmonic multiple loop detection algorithm for vital sign monitoring. Duan et al. [28] used the variational mode decomposition algorithm and applied the Hilbert transform to extract time–frequency information from vital signs. Ren et al. [29] applied a moving target indicator filter to the UWB signals and utilized an FFT to find the highest frequency of the spectrogram. The algorithms in these studies involved the process of removing respiratory harmony components that affect HR components. In this study, a vital estimation model was constructed to extract the relationship between RR and HR automatically without removing the harmonic components. To check whether the information about the relationship between RR and HR was sufficiently learned, a model that estimates only respiration or HR was additionally trained, and its performance was compared with that of the vital estimation model (Table 4).

**Figure 6:**
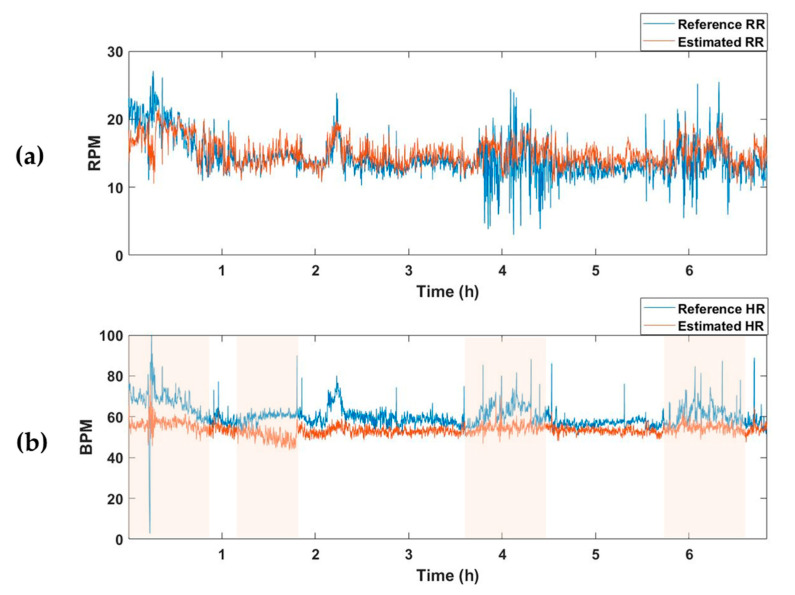
Vital-sign detection results obtained using the RR and HR estimation models. (**a**) Reference RR and estimated RR from RR predictor, and (**b**) reference HR and estimated HR from HR predictor. Pink shaded areas indicate the sections in which the MAE was larger than the HR estimated using the vital predictor in Figure 5. RPM, respiration per minute; BPM, beats per minute.

The average MAE value for the test set of the model trained to estimate only respiration was 2.63, and there was no significant difference from the RR-estimated MAE value of 2.67 in the vital estimation model. On the other hand, after training a model that estimates only the HR, the average MAE for the test set was 7.49, which was higher than the HR-estimated MAE of the vital estimation model, 4.78. In other words, when the model is trained to estimate two vital signs simultaneously rather than extracting only one vital sign, information about the relationship between the two vital signs is also trained, resulting in better estimation performance. Figure 5 and Figure 6 are examples of the estimation results obtained by applying the model estimating two vital signs and the model estimating only one vital sign (RR or HR) to the one subject recording of a test dataset. Although there was no significant difference in the estimation of RR (Figure 5a and Figure 6a), the HR detection performance was worse when the model trained only estimating HR, as can be seen in the pink shaded areas (Figure 5b and Figure 6b).

Further, as the length of window size increased, the estimation performance improved. If the window size is 5 s, a respiratory rate of less than two cycles is normally included. Thus, the model may not have extracted a significant filter because respiration pattern information was not sufficient. When the window was increased, we confirmed that the respiration cycle was more included in the segment, and the model learned more information, improving vital-sign estimation performance. In this study, motion and noise artifacts were extracted, and this section was postprocessed when estimating the final vital signs. The estimated vital-sign values were stored in a buffer with a size of 5 s, and the vital-sign values were updated with the average value of the buffer in the artifact sections. From this postprocessing, it is possible to improve the rapid change of vital signs in the artifacts section. We confirmed that the average RR MAE of the test dataset decreased from 2.77 to 2.67 and the average HR MAE decreased from 5.01 to 4.78.

In this study, we conducted several additional experiments to validate the performance of our proposed model. Firstly, we examined the performance when adding an LSTM layer to the back of the CNN model. Two bidirectional LSTM layers were used, and the hidden unit was set to 128. We assumed that the CNN model could extract sufficient locality information from the input data and that the impact of time dependency information decreased as the short-length input data passed through the CNN layer. As shown in Table 5, the average RR and HR estimation performance decreased to 3.12 and 5.26, respectively. This result indicates that locality information extracted from the CNN model is more significant than time-dependent information. Second, we investigated the performance difference between building a model as scratch method proposed method in this paper and fine-tuning method using the pretrained model. We examined the performance of the vital-sign estimation for 2D input images by fine-tuning representative models in image classifiers such as Resnet [32], Densenet [33], Googlenet [34], and Vision Transformer [35]. Although the number of model parameters was considerably higher compared to that in the proposed model, there was no significant difference in performance (Table 5). To estimate vital signs in real time, the complexity of the model is also one of the important factors, and from this perspective, the model proposed in this study was better than the existing pretrained model. Lastly, we augmented the dataset by dividing the 2D signal into bands and training the model. CWT images of the original 0–5 Hz band were divided into three segments to increase the amount of data by three times, and training was conducted. The results show that the MAE of the model increased to 2.97 and 5.11 for RR and HR, respectively. The reason for the decrease in performance despite the increase in data is that the original model could learn features related to harmonic components and interactions in the entire frequency range of 0–5 Hz, whereas dividing the frequency range into segments could cause the loss of such information, resulting in worse performance.

When we evaluated vital-sign estimation performance, the test dataset included SAHS subjects with AHIs of 30 or higher. As can be seen in Table 3, the RR MAE values of subjects with high AHIs were higher than those with low AHIs. One of the reasons for this result is that the vital-sign estimation model was trained on the basis of data with normal breathing. To improve the estimation performance, a model including the segments of apnea–hypopnea must be trained, and the model must be combined with the AH event detection algorithm. Furthermore, the average HR MAE of the test dataset was higher than the average RR MAE of the test dataset, which may have been affected by the location and noise of the IR-UWB radar. Previous studies [11,12,13,14,19,29] obtained data after installing the radar direction to face the front of the chest; however, in this study, considering its use in an actual environment, the radar was diagonally oriented rather than facing the chest of the subject. In addition, the distance and blanket during sleep may affect the HR estimation performance. To improve the HR estimation performance further, the radar installation angles and distances need to be adjusted, and increasing the impulse center frequency to detect more sensitive heartbeat can be a means of achieving this objective.

Most existing studies designed and validated algorithms on the basis of data measured in a static state for a short time; the significance of this study lies in the fact that it verified vital-sign estimation performance after measuring radar in an environment that included dynamic situations for a long time. A total of 13,245 min of data of the segments was used in the entire analysis. When the radar signals are measured during the whole night of sleep, the artifacts due to dynamic movement affects vital-sign estimation. Therefore, in this study, motion and noise components were detected, and postprocessing was performed for the artifact section. As a result, RR and HR estimation performance was improved.

Although meaningful results were obtained in this study, it suffers from a few limitations. First, the proposed vital predictor model was trained and validated on the basis of the data of subjects with normal breathing. The proposed model should be verified for a person with a breathing-related disease in which apnea and hypopnea events occur during sleep. Second, assuming a utilization situation in the home environment, data were obtained after radar installation; however, they were measured together when performing PSG inspection in a controlled laboratory environment and not in the actual home environment. To examine the feasibility of the proposed model, it must be verified after obtaining real-world data. Third, several preprocessing steps were performed before inputting the data into the DL model. To simplify the preprocessing further, additional research is needed to generalize the index search and artifact detection using DL models. Lastly, further studies need to investigate the model complexity for application to embedded and IoT systems.

## 5. Conclusions

In this study, a CNN-based deep learning framework was developed for real-time vital-sign monitoring using a noncontact IR-UWB radar. The position of the subject was searched on the basis of STD values, and 1D and 2D signals applied with CWT were used as inputs to the CNN models, which were subsequently concatenated to estimate the vital-sign values in real time. The results showed that, when trained to extract both RR and HR, the model performed better than when extracting only one value; further increasing the number of layers also improved the estimation performance, and a window size of 15 s yielded the best results. Thus, our model provided competitive results in estimating long-term vital signs including both dynamic and static states. IR-UWB radar-based long-term vital-sign monitoring provides valuable information about the health status and can help in preventing or managing potential health problems.

## Figures and Tables

**Table 1:** Summary of sleep-related variables and subject characteristics (mean ± standard deviation, S.D.).

Variables	Mean ± S.D.
Sex (male/female)	14/16
Age (years)	27.2 ± 4.7
BMI (kg/m^2^)	21.9 ± 3.6
AHI (events/h)	5.8 ± 11.9
Total recording time (min)	441.5 ± 33.5
Total sleep time (min)	408.7 ± 41.4
Sleep onset latency (min)	7.8 ± 10.5
Sleep efficiency (%)	92.5 ± 4.6

S.D., standard deviation; BMI, body mass index; AHI, apnea–hypopnea index.

**Table 2:** Configuration summary of the proposed model.

Layer Type	Filter Size@ Kernel Size	Activation Function	Output Shape	Number of Parameters
Input 1D	1 × 300			
Conv 1D_1	128 @ 1 × 3	ReLu	1 × 149	512
BatchNorm 1D_1			1 × 149	256
Max pool 1D_1	1 × 2		1 × 148	
Conv 1D_2	128 @ 1 × 3	ReLu	1 × 73	49,280
BatchNorm 1D_2			1 × 73	256
Max pool 1D_2	1 × 2		1 × 72	
Conv 1D_3	128 @ 1 × 3	ReLu	1 × 35	
BatchNorm 1D_3			1 × 35	49,280
Max pool 1D_3	1 × 2		1 × 34	256
Input 2D	123 × 300			
Conv 2D_1	128 @ 3 × 3	ReLu	61 × 149	
BatchNorm 2D_1			61 × 149	1280
Max pool 2D_1	2 × 2		60 × 148	256
Conv 2D_2	128 @ 3 × 3	ReLu	29 × 73	
BatchNorm 2D_2			29 × 73	147,584
Max pool 2D_2	2 × 2		28 × 72	256
Conv 2D_3	128 @ 3 × 3	ReLu	13 × 35	
BatchNorm 2D_3			13 × 35	147,584
Max pool 2D_3	2 × 2		12 × 34	256
1D FC_1		ReLu	4352	43,530
2D FC_2		ReLu	52,224	522,250
FC_3			10	
Output			2	42

**Table 3:** MAE test results for each subject.

Subjects	AHI	MAE of RR	MAE of HR
1	10.4	1.91	3.76
2	8.1	1.83	4.96
3	50.3	4.59	5.97
4	45.9	6.76	3.63
5	17.5	4.29	4.26
6	1.1	1.91	6.19
7	3.6	1.96	4.88
8	4.4	1.82	4.50
9	5.7	2.65	5.02
10	0.0	1.71	4.58
11	0.5	3.55	3.95
12	0.2	1.57	5.25
13	0.9	1.55	6.00
14	0.5	1.93	3.80
15	1.5	1.94	4.85
**AVG**	**10.0**	**2.67**	**4.78**
**STD**	**15.7**	**1.45**	**0.80**

MAE, mean absolute error; AHI, apnea–hypopnea index; RR, respiration rate; HR, heart rate; AVG, average; STD, standard deviation.

**Table 4:** Vital signs detection results of RR, HR, and vital estimation model on the test dataset (mean ± STD of MAE).

RR Estimation Model	HR Estimation Model	Vital Estimation Model	
		RR	HR
2.63 ± 1.36	7.49 ± 1.56	2.67 ± 1.45	4.78 ± 0.80

MAE, mean absolute error; RR, respiration rate; HR, heart rate.

**Table 5:** Vital-sign detection performance using the proposed model and pretrained models on the test dataset. (mean ± STD of MAE).

	Estimation Performance		
	RR	HR	Number of Parameters
**Proposed Model**	**2.67 ± 1.45**	**4.78 ± 0.80**	**522,250**
CNN + LSTM (1D + 2D input)	3.12 ± 1.55	5.26 ± 0.95	3,160,382
GoogleNet (2D input)	2.45 ± 1.41	4.93 ± 0.67	6,626,906
ResNet (2D input)	2.83 ± 1.51	4.70 ± 0.75	11,691,514
DenseNet (2D input)	2.84 ± 1.49	4.81 ± 0.75	28,683,002
Vision Transformer (2D input)	2.28 ± 1.21	8.09 ± 3.88	86,569,658

CNN, convolutional neural networks; LSTM, long short-term memory; MAE, mean absolute error; RR, respiration rate; HR, heart rate.

## Data Availability

Not applicable.
